# Time in range and complications of diabetes: a cross-sectional analysis of patients with Type 1 diabetes

**DOI:** 10.1186/s13098-023-01219-2

**Published:** 2023-11-27

**Authors:** Marta Fernandes Bezerra, Celestino Neves, João Sérgio Neves, Davide Carvalho

**Affiliations:** 1 Faculty of Medicine of the University of Porto, Alameda Hernâni Monteiro, Porto, 4200-319 Portugal; 2 Department of Endocrinology, Diabetes and Metabolism, Centro Hospitalar Universitário de São João, Porto, Portugal; 3 Instituto de Investigação e Inovação em Saúde, Universidade do Porto, Porto, Portugal; 4 Cardiovascular R&D Centre - UnIC@RISE, Department of Surgery and Physiology, Faculty of Medicine of the University of Porto, Porto, Portugal

**Keywords:** Diabetes complications, Time in range, HbA1c, Type 1 diabetes

## Abstract

**Background/ objective:**

To evaluate the association of CGM parameters and HbA1c with diabetes complications in patients with Type 1 Diabetes (T1D).

**Methods:**

Patients with T1D using the CGM system Freestyle Libre were included in this analysis. The association of CGM-metrics and HbA1c with diabetes complications (any complication, microvascular complications, or macrovascular complications) was assessed using logistic regression unadjusted and adjusted for age, sex, and diabetes duration (model 1), and further adjusted for hypertension and dyslipidemia (model 2).

**Results:**

One hundred and sixty-one patients with T1D were included. The mean (± SD) age was 37.4 ± 13.4 years old and the median T1D duration was 17.7 ± 10.6 years. Time in range (TIR) was associated with any complication and microvascular complications in the unadjusted model and in the adjusted models. TIR was associated with retinopathy in the unadjusted model as well as in model 1, and was associated with macrovascular complications only in the unadjusted model. HbA1c was associated with any complications, microvascular complications, and retinopathy in the unadjusted model but not in the adjusted models. HbA1c was associated with macrovascular complications in the unadjusted model and in the adjusted model 1.

**Conclusions:**

In this cross-sectional analysis of patients with T1D using intermittent scanned CGM, TIR, and HbA1c were associated with complications of diabetes. TIR may be a better predictor than HbA1c of any complication and microvascular complications, while HbA1c may be a better predictor of macrovascular complications.

**Supplementary Information:**

The online version contains supplementary material available at 10.1186/s13098-023-01219-2.

## Background/ introduction

Hemoglobin A1c (HbA1c) is one of the most used biomarkers to monitor blood glucose levels, as it is reliable, relatively easy, and cheap to obtain. Thus, HbA1c is generally accepted as the gold standard to assess blood glucose control [1–3]. It is well-established that HbA1c levels are associated with the development of complications of diabetes [2, 4, 5]. However HbA1c has several limitations in clinical practice [3, 6]. First, HbA1c reflects the glycemia of the last 2–3 months and, as such, does not provide continuous feedback for the optimization of glycemic control [3, 6]. Second, HbA1c is not accurate in several conditions including in patients with anemia, iron deficiency, hemoglobinopathies, or chronic kidney diseases [3, 6, 7]. Finally, HbA1c does not reflect glycemic variability [8]. In patients with frequent hypoglycemia, HbA1c may underestimate the simultaneous burden of hyperglycemia [8].

Due to the limitations of HbA1c, continuous glucose monitoring (CGM) devices have been recommended as a central tool for managing patients with Type 1 Diabetes (T1D) [9]. Time in range (TIR, percentage of time glucose is within the range of 70–180 mg/dL) is one of the main parameters obtained from CGM [10–12]. TIR correlates well with HbA1c levels, and a TIR of 65–70% corresponds to an HbA1c level of 6.5–7% [13, 14]. TIR can be continuously assessed by the patients as well as the clinician and, as such, is a measure with more clinical utility than HbA1c. However, whether TIR is associated with microvascular and macrovascular complications of diabetes is still uncertain [15, 16]. Furthermore, whether other metrics of CGM (time above range, time below range, glycemic variability) are associated with complications in T1D is also not settled [16].

We hypothesized that TIR is associated with microvascular and macrovascular complications in a cross-sectional analysis of patients with T1D. The main objective of this study was to evaluate the association of CGM parameters and HbA1c with complications of diabetes in T1D.

## Materials and methods

### Study design

This cross-sectional study was conducted at Centro Hospitalar Universitário de São João (CHUSJ), Porto, Portugal. We included patients with T1D followed in the outpatient clinic between 2020 and 2022 using the intermittent scanned CGM system Freestyle Libre (is-CGM). This study was approved by the Ethical Committee of CHUSJ.

Clinical data was collected from electronic health records and is-CGM data was collected from the cloud-based system LibreView.

### Inclusion and exclusion criteria

We included patients aged 18 years or older with T1D using the CGM system Freestyle Libre. Participants were identified from the list of patients associated with CHUSJ in the LibreView platform. We excluded repeated accounts, patients with other types of diabetes, patients without is-CGM data uploads, patients without HbA1c evaluations, and patients without active sensor time of at least 70% at the time of HbA1c evaluation (Supplementary Fig. 1).

### Clinical data

The following parameters were obtained from the electronic health records: gender, age, and education (less than high school graduate; high school graduate; some college education; college degree or higher), duration of diabetes, hypertension (defined as previously diagnosed hypertension or treatment with blood pressure lowering drugs), systolic and diastolic blood pressure, dyslipidemia (defined as a previous diagnosis, under lipid-lowering drugs); type of diabetes treatment (insulin pens or insulin pumps); use of other antidiabetic drugs; anthropometric parameters (weight, height, and body mass index). HbA1c levels and lipid profiles (total cholesterol, LDL cholesterol, HDL cholesterol, and triglycerides) were obtained from routine laboratory evaluations.

### CGM data

The following parameters were obtained from the LibreView platform (data from 14 days): time CGM is active, TIR (70–180 mg/dL), time below range (TBR, < 70 mg/dL), time below 54 mg/dL (TB54), time above range (TAR, > 180 mg/dL), time above 250 mg/dL (TA250), coefficient of variation (CV) and glucose management indicator (GMI).

### Complications of diabetes

Complications of diabetes were obtained from electronic health records. Any complication was defined as having microvascular or macrovascular complication.

The following microvascular complications were collected: retinopathy (defined as any degree of retinopathy in ophthalmologic evaluation), nephropathy (defined as a confirmed value of albumin to creatinine ratio of 30 mg/g or greater, or eGFR of less than 60 mL/min/1.73 m^2^), diabetic peripheral neuropathy (defined by a change in the assessment of 10-g monofilament testing or changes in temperature or pinprick sensation). The following macrovascular complications were collected: ischemic coronary disease (defined as a history of myocardial infarction, stable or unstable angina, or myocardial revascularization), cerebrovascular disease (characterized as a history of stroke or transient ischemic attack) and peripheral arterial disease (defined as a history of intermittent claudication, ankle-brachial index ≤ 0.90 or lower-extremity revascularization). Heart failure diagnosis was also assessed (defined by clinical diagnosis of heart failure or previous hospitalization due to the same pathology).

### Statistical analysis

Continuous variables are described as mean ± standard deviation and categorical variables as proportions (percentages).

The associations of CGM-metrics and HbA1c with any complication, microvascular complications, and macrovascular complications were assessed by logistic regression unadjusted and adjusted for age, sex, and duration of diabetes (model 1), and further adjusted for hypertension and dyslipidemia (model 2). For the individual components of macrovascular complications and diabetic peripheral neuropathy, only the unadjusted analysis was performed due to the low number of events.

All analyses were conducted with the statistical software package Stata IC version 17.0 (College Station, TX). A two-sided *P*-value < 0.05 was considered statistically significant.

## Results

A total of 161 patients (Supplementary Fig. 1) were included in the present analysis. 60% were male, the mean age was 37.4 ± 13.4 years old, the mean diabetes duration was 17.7 ± 10.6 years and 40.6% percent were using an insulin pump (all patients were using stand-alone systems). 19% had hypertension and 41.1% had dyslipidemia. In this population, 52 patients (32.3%) had at least one complication. Forty-nine (30.4%) had microvascular complications and 13 (8.1%) had macrovascular complications. The most common microvascular complication was retinopathy (39 patients, 24.2%), followed by nephropathy (18 patients, 11.2%) and diabetic peripheral neuropathy (4 patients, 2.5%). No patient had heart failure (Table 1).

**Table 1:** Characteristics of the study sample (n = 161)

Male sex, %	97 (60.2%)
Age, years	37.4 ± 13.4
Duration of diabetes, years	17.7 ± 10.6
Insulin pump, %	65 (40.6%)
Hypertension, %	30 (18.9%)
Dyslipidemia, %	65 (41.1%)
Body mass index, kg/m^2^	24.8 ± 4.0
Systolic pressure, mmHg	126.0 ± 15.5
Diastolic pressure, mmHg	72.6 ± 10.0
Total cholesterol, mg/dL	164.5 ± 34.6
LDL cholesterol, mg/dL	93.6 ± 26.9
HDL cholesterol, mg/dL	57.0 ± 14.5
Triglycerides, mg/dL	78.1 ± 38.0
Education	
Less than hight school graduate, %	1 (1.5%)
High school graduate, %	1 (1.5%)
Some college education, %	23 (35.4%)
College degree or higher, %	40 (61.5%)
Other antidiabetic drugs	
SGLT2 inhibitors, %	18 (11.2%)
Metformin, %	17 (10.6%)
GLP-1 analogue, %	5 (3.1%)
DPP-4 inhibitors, %	1 (0.6%)
Thiazolidinedione, %	0 (0%)
Sulfonylureas, %	0 (0%)
Any complication	52 (32.3%)
Microvascular complication	49 (30.4%)
Retinopathy, %	39 (24.2%)
Nephropathy, %	18 (11.2%)
Peripheric neuropathy, %	4 (2.5%)
Macrovascular complication	13 (8.1%)
Ischemic coronary disease, %	4 (2.5%)
Cerebrovascular disease, %	5 (3.1%)
Peripheral arterial disease, %	6 (3.7%)
Heart Failure	0 (0%)

Categorical variables are presented as counts (percentages). Continuous variables are presented as mean ± standard deviation. LDL: low-density lipoprotein. HDL: High-density lipoprotein. SGLT2: Sodium-glucose Cotransporter-2. GLP-1: Glucagon-like peptide-1. DPP4: Inhibitors of dipeptidyl peptidase 4

Table 2 shows the CGM-metrics and HbA1c level. The mean TIR was 57.5 ± 17.3%, the mean TBR was 4.9 ± 4.3% and the mean CV was 38.7 ± 7.4%. The mean HbA1c level was 7.5 ± 1.1%.

**Table 2:** HbA1c levels and CGM-metrics (n = 161)

HbA1c, %	7.5 ± 1.1
Amount of time CGM is active, %	90.6 ± 9.4
TIR, %	57.5 ± 17.3
TBR, %	4.9 ± 4.3
TB54, %	1.1 ± 2.1
TAR, %	37.5 ± 18.2
TA250, %	13.9 ± 12.6
CV, %	38.7 ± 7.4
GMI, %	7.3 ± 0.8

Categorical variables are presented as counts (percentages). Continuous variables are presented as mean ± standard deviation. CGM: continuous glucose monitoring. TIR: Time in range, 70–180 mg/dL. TB54: Time below 54 mg/dL. TBR: Time below range, 70 mg/dL. TA250: Time above 250 mg/dL. TAR: Time above range, 180 mg/dL. CV: Glucose variability, defined as the percentage coefficient of variation. GMI: glucose management indicator), is a parameter derived from the measured glucose levels. HbA1c: glycated hemoglobin

Table 3 shows the association of CGM-metrics and HbA1c with diabetes complications. TIR was associated in the unadjusted and adjusted models with any complication (odds ratio, OR, 0.56, 95%CI, 0.37–0.87 per 10% increase in model 2) and microvascular complications (OR 0.63, 95%CI, 0.41–0.95 per 10% increase in model 2). TIR was associated with retinopathy in the unadjusted model and in model 1, and was associated with macrovascular complications only in the unadjusted model. HbA1c was associated with any complication in the unadjusted model but not in the adjusted models (OR 1.32, 95%CI, 0.88–1.99 per 1% increase in model 2). HbA1c was also associated with microvascular complications and retinopathy only in the unadjusted model. HbA1c was associated with macrovascular complications in the unadjusted model and in the adjusted model 1 (OR 1.82, 95%CI, 1.11-3.00 per 1% increase), but not in the model further adjusted for hypertension and dyslipidemia (OR 1.59, 95%CI, 0.93–2.73 per 1% increase in model 2).

**Table 3:** Association of HbA1c levels and CGM-metric with diabetes complications

Outcomes	Odds ratio (95% CI)	*P* Value	Odds ratio (95% CI)	*P* Value	Odds ratio (95% CI)	*P* Value	Odds ratio (95% CI)	*P* Value
	**HbA1c** **(%)**		**TIR** **(per 10% increase)**		**TB54** **(%)**		**TBR** **(%)**	
Any complication								
Unadjusted	1.49 (1.09–2.04)	**0.012**	0.70 (0.56–0.86)	**0.001**	1.13 (0.96–1.32)	0.132	1.01 (0.94–1.09)	0.737
Model 1	1.27 (0.89–1.82)	0.181	0.70 (0.53–0.93)	**0.012**	0.99 (0.83–1.19)	0.941	0.98 (0.90–1.07)	0.666
Model 2	1.32 (0.88–1.99)	0.183	0.56 (0.37–0.87)	**0.009**	0.98 (0.72–1.34)	0.922	0.92 (0.80–1.06)	0.248
Microvascular complications								
Unadjusted	1.43 (1.05–1.94)	**0.023**	0.71 (0.58–0.88)	**0.002**	1.08 (0.93–1.26)	0.311	1.01 (0.93–1.09)	0.867
Model 1	1.22 (0.85–1.75)	0.288	0.72 (0.54–0.97)	**0.028**	0.94 (0.78–1.12)	0.475	0.97 (0.88–1.06)	0.465
Model 2	1.26 (0.83–1.92)	0.271	0.63 (0.41–0.95)	**0.029**	1.02 (0.74–1.39)	0.915	0.96 (0.83–1.10)	0.543
Retinopathy								
Unadjusted	1.49 (1.08–2.06)	**0.015**	0.73 (0.58–0.91)	**0.006**	1.08 (0.92–1.27)	0.345	0.98 (0.89–1.07)	0.609
Model 1	1.32 (0.91–1.90)	0.139	0.74 (0.55-1.00)	**0.047**	0.98 (0.81–1.18)	0.823	0.95 (0.86–1.05)	0.356
Model 2	1.32 (0.86–2.01)	0.202	0.77 (0.52–1.14)	0.195	1.25 (0.89–1.76)	0.201	1.01 (0.88–1.17)	0.846
Nephropathy								
Unadjusted	1.23 (0.84–1.80)	0.281	0.84 (0.63–1.12)	0.234	1.01 (0.80–1.27)	0.948	1.02 (0.91–1.14)	0.774
Model 1	1.07 (0.67–1.70)	0.783	0.94 (0.66–1.32)	0.704	0.94 (0.73–1.20)	0.612	1.01 (0.90–1.13)	0.923
Model 2	1.22 (0.56–2.66)	0.623	0.69 (0.39–1.22)	0.200	0.81 (0.55–1.19)	0.281	0.90 (0.75–1.08)	0.273
Macrovascular complications								
Unadjusted	1.85 (1.20–2.85)	**0.006**	0.66 (0.46–0.93)	**0.019**	1.10 (0.88–1.38)	0.388	0.93 (0.80–1.09)	0.393
Model 1	1.82 (1.11-3.00)	**0.018**	0.65 (0.41–1.04)	0.070	1.10 (0.86–1.40)	0.445	0.97 (0.82–1.15)	0.709
Model 2	1.59 (0.93–2.73)	0.090	0.68 (0.39–1.16)	0.152	0.92 (0.62–1.34)	0.652	0.77 (0.54–1.11)	0.166
	**TAR** **(%)**		**TA250** **(%)**		**CV** **(%)**		**GMI** **(%)**	
Any complication								
Unadjusted	1.03 (1.01–1.05)	**0.002**	1.03 (1.01–1.06)	**0.017**	1.05 (1.00-1.10)	**0.039**	1.79 (1.16–2.75)	**0.009**
Model 1	1.03 (1.00-1.06)	**0.020**	1.03 (1.00-1.07)	0.082	1.03 (0.97–1.09)	0.350	1.69 (0.96–2.97)	0.069
Model 2	1.05 (1.01–1.09)	**0.011**	1.04 (1.00-1.09)	0.056	1.00 (0.91–1.09)	0.929	2.27 (1.08–4.73)	**0.029**
Microvascular complications								
Unadjusted	1.03 (1.01–1.05)	**0.003**	1.03 (1.00-1.06)	**0.026**	1.05 (1.00-1.10)	0.056	1.75 (1.14–2.71)	**0.011**
Model 1	1.03 (1.00-1.06)	**0.036**	1.03 (0.99–1.06)	0.117	1.02 (0.96–1.09)	0.481	1.66 (0.93–2.96)	0.086
Model 2	1.04 (1.00-1.08)	**0.045**	1.04 (0.99–1.08)	0.100	1.02 (0.93–1.11)	0.664	1.96 (0.96–4.02)	0.065
Retinopathy								
Unadjusted	1.03 (1.01–1.05)	**0.004**	1.03 (1.00-1.06)	**0.043**	1.03 (0.98–1.08)	0.230	1.81 (1.15–2.85)	**0.011**
Model 1	1.03 (1.00-1.06)	**0.037**	1.02 (0.99–1.06)	0.179	1.00 (0.94–1.07)	0.903	1.67 (0.94–2.98)	0.083
Model 2	1.02 (0.99–1.06)	0.236	1.03 (0.98–1.07)	0.251	1.00 (0.91–1.09)	0.951	1.55 (0.77–3.14)	0.223
Nephropathy								
Unadjusted	1.01 (0.99–1.04)	0.281	1.02 (0.98–1.05)	0.361	1.06 (0.99–1.14)	0.084	1.32 (0.74–2.37)	0.348
Model 1	1.00 (0.97–1.04)	0.796	1.01 (0.96–1.05)	0.806	1.05 (0.97–1.13)	0.244	1.04 (0.52–2.05)	0.914
Model 2	1.04 (0.99–1.09)	0.170	1.01 (0.95–1.07)	0.702	1.02 (0.91–1.14)	0.736	1.62 (0.60–4.37)	0.339
Macrovascular complications								
Unadjusted	1.04 (1.01–1.08)	**0.010**	1.04 (1.00-1.08)	**0.029**	1.03 (0.95–1.11)	0.524	2.17 (1.14–4.11)	**0.018**
Model 1	1.04 (1.00-1.08)	0.070	1.04 (0.99–1.09)	0.154	1.01 (0.91–1.11)	0.901	1.78 (0.80–3.96)	0.155
Model 2	1.04 (0.99–1.10)	0.083	1.03 (0.97–1.09)	0.286	0.92 (0.81–1.06)	0.252	2.03 (0.77–5.37)	0.154

Model 1: adjusted to age, sex, and duration of diabetes Model 2: adjusted to age, sex, and duration of diabetes, hypertension and dyslipidemia. CGM: continuous glucose monitoring. TIR: Time in range, 70–180 mg/dL. TB54: Time below 54 mg/dL. TBR: Time below range, 70 mg/dL. TA250: Time above 250 mg/dL. TAR: Time above range, 180 mg/dL. CV: Glucose variability, defined as the percentage coefficient of variation. GMI: glucose management indicator), is a parameter derived from the measured glucose levels. HbA1c: glycated hemoglobin

Regarding other CGM-metrics, in the adjusted model 2, GMI was associated with any complication and TAR was associated with any complication and microvascular complication. Of note, CV was associated with any complication in the unadjusted model but not in the adjusted models, and was not significantly associated with other outcomes.

The association of CGM-metrics and HbA1c with individual components of macrovascular complications and diabetic peripheral neuropathy (complications with low prevalence in this population) are shown in supplementary Table 1.

## Discussion

In this cross-sectional analysis of patients with T1D using is-CGM, TIR was associated with the presence of any complication and with microvascular complications. On the other hand, the association of HbA1c with any complication was not significant after adjustment for confounders. Interestingly, HbA1c was associated with macrovascular complications even after adjustment for age, sex, and diabetes duration, while TIR was only associated with macrovascular complications in the unadjusted analysis.

As seen in other studies, HbA1c and TIR are associated with diabetes complications. However, most previous studies were focused on patients with type 2 diabetes (T2D) and evaluated only a few days of CGM data [16]. In a study of 3262 patients with T2D, Lu J et al. found a significant association between lower TIR and a higher prevalence of diabetic retinopathy [17]. Yoo JH et al. described that in a population of 866 subjects the TIR and hyperglycemia metrics were strongly associated with albuminuria in type 2 diabetes [18]. Our results are concordant with the finding of an increased risk of microvascular complications in patients with lower TIR. The lack of association of TIR and other metrics with nephropathy is probably related to the lower statistical power of our study [18].

Regarding studies in patients with T1D, one study using the DCCT data found that TIR had an association with the risk of development and progression of retinopathy [15]. However, in this study the TIR was derived from 7 finger-prick samples and not from CGM use. One of the largest studies using CGM in T1D was the study by Malahi AE et al. in 515 Belgian adults with T1D using sensor-augmented pump therapy [19]. In this study, lower TIR and higher HbA1c levels were associated with microvascular complications. Consistent with our findings, there was no association between CV and complications of diabetes, and macrovascular complications were associated with HbA1c levels but not with TIR [19]. Although our study had a smaller population, we included not only participants using insulin pumps but also patients treated with insulin pens. Furthermore, in the study by Malahi AE et al., the data from CGM metrics were obtained from the first 2 weeks of RT-CGM (real-time continuous glucose monitoring) use, which may not represent the usual glucose control of the studied population, since with the prolonged use of CGM there is an improvement in glycemic control. In our study, the data were obtained from the routine use of is-CGM. Whether our results also apply to RT-CGM is uncertain. At the time of our study, most patients were already using Freestyle Libre 2 which allows the activation of alarms for hypoglycemia and hyperglycemia. The availability of alarms may decrease the clinical differences between is-CGM and RT-CGM. Our study, together with the evidence previously described, supports the relevance of the use of CGM to assess glycemic control and predict diabetes complications. Our data raises the hypothesis that TIR may be a better predictor of any complication and microvascular complications than HbA1c. As hyperglycemia is the main mechanism driving the risk of microvascular complications [20, 21] and TIR better reflects the glycemic control than HbA1c (which may be influenced by several clinical conditions and may be falsely decreased by hypoglycemia), it is plausible that TIR is a better marker for predicting microvascular complications. Given the cross-sectional design of our study, this hypothesis must be interpreted with caution and requires confirmation from prospective studies. Interestingly, for macrovascular complications, HbA1c may be a better predictor than TIR according to our results (Table 3). Previous studies have suggested that the hemoglobin glycation index (the difference between the measurement of HbA1c and mean plasma glucose level) may be an independent predictor of cardiovascular disease, [22, 23] which would explain the added value of using HbA1c to predict macrovascular complications. Furthermore, other mechanisms may be implicated in this association and we cannot exclude that this association could be a result of the limited statistical power or due to chance. More studies are necessary to better clarify this association.

Our study has limitations that we must acknowledge. First, HbA1c and CGM metrics were assessed in a single moment for each patient, not taking into account potential variations over time. Second, our population was young (mean age of 37 years) and with a low prevalence of nephropathy and macrovascular complications which may have decreased our ability to detect significant associations. Third, despite the use of two different models of adjustment to try to address confounding, we cannot exclude the possibility of residual confounders (e.g. lifestyle parameters including exercise and diet were not evaluated). Finally, the limitations of our study design should be taken into consideration. As a cross-sectional analysis, we cannot evaluate the association of HbA1c and TIR with future complications. Regarding the strengths of our study, we must highlight that we evaluate patients with T1D which was not done in most previous studies [16]. Contrary to several previous reports that used only 2 to 3 days of CGM, we evaluated the 14 days which is the recommended duration for clinical practice [9, 16]. However, it should be noted that although a 14-day period is the recommended timeframe for analysis, it might not always fully represent all glycemic metrics. This is primarily due to the substantial variations observed from week to week in certain parameters, particularly TBR [24].

Furthermore, the period in which CGM metrics and HbA1c levels were evaluated were matched (only 7 days difference was allowed) which may allow a better comparison of the value, of each of these tools, to predict the risk of diabetes complications.

## Conclusions

In conclusion, in this cross-sectional analysis of patients with T1D using is-CGM, TIR, and HbA1c were associated with complications of diabetes. After adjustment for confounder TIR was associated with any complication and microvascular complications, while was associated with macrovascular complications. Further studies, ideally prospective studies, to confirm our findings and to better characterize the role of CGM-metrics and HbA1c to predict long-term complications in diabetes.

### Electronic supplementary material

Below is the link to the electronic supplementary material.

Supplementary Material 1

## Data Availability

The datasets used and/or analysed during the current study are available from the corresponding author on reasonable request.

## List of abbreviations

T1D: Type 1 Diabetes
TIR: Time in range
HbA1c: Hemoglobin A1c
CGM: continuous glucose monitoring
Is-CGM: intermittent scanned CGM
CHUSJ: Centro Hospitalar Universitário de São João
TBR: time below range
TB54: time below 54 mg/dL
TAR: time above range
TA250: time above 250 mg/dL
CV: coefficient of variation
GMI: glucose management indicator
OR: odds ratio
T2D: type 2 diabetes
RT-CGM: real-time continuous glucose monitoring
