# Supplementary material for: Time in range and complications of diabetes: a cross-sectional analysis of patients with Type 1 diabetes
Source: Diabetol Metab Syndr. 2023 Nov 27;15:244. doi: 10.1186/s13098-023-01219-2 (PMC10680248; doi:10.1186/s13098-023-01219-2)
Supplement: Supplementary file 1 — Supplementary Material 1 [file 13098_2023_1219_MOESM1_ESM.docx]

| **Supplementary Table 1.** | | | | | | | | |
| --- | --- | --- | --- | --- | --- | --- | --- | --- |
|  | HbA1c  (%) | | TIR  (per 10% incresse) | | TB54  (%) | | TBR  (%) | |
|  | Odds ratio  (95% CI) | P Value | Odds ratio  (95% CI) | P Value | Odds ratio  (95% CI) | P Value | Odds ratio  (95% CI) | P Value |
| **Outcomes** |  |  |  |  |  |  |  |  |
| Ischemic coronary disease | 1.92 (1.11-3.33) | **0.020** | 0.55 (0.29-1.02) | 0.057 | 1.18 (0.86-1.63) | 0.295 | 0.98 (0.76-1.25) | 0.851 |
| Cerebral vascular disease | 1.63 (0.97-2.74) | 0.066 | 0.58 (0.33-1.01) | 0.053 | 1.15 (0.85-1.56) | 0.356 | 0.94 (0.74-1.21) | 0.634 |
| Arterial Peripheric disease | 1.51 (0.91-2.50) | 0.111 | 0.76 (0.47-1.23) | 0.268 | 0.59 (0.20-1.77) | 0.346 | 0.77 (0.54-1.08) | 0.134 |
| Peripheral Neuropathy | 1.67 (0.96-2.90) | 0.068 | 0.60 (0.33-1.10) | 0.101 | 0.49 (0.09-2.54) | 0.395 | 0.62 (0.35-1.12) | 0.113 |
|  | TAR  (%) | | TA250  (%) | | CV  (%) | | GMI  (%) | |
|  | Odds ratio (95% CI) | P Value | Odds ratio (95% CI) | P Value | Odds ratio (95% CI) | P Value | Odds ratio 95% CI) | P Value |
| **Outcomes** |  |  |  |  |  |  |  |  |
| Ischemic coronary disease | 1.05 (1.00-1.11) | 0.066 | 1.05 (0.99-1.11) | 0.114 | 1.05 (0.92-1.20) | 0.471 | 2.47 (0.92-6.68) | 0.074 |
| Cerebral vascular disease | 1.06 (1.00-1.11) | **0.032** | 1.05 (0.99-1.11) | 0.084 | 0.98 (0.87-1.11) | 0.754 | 2.22 (0.89-5.55) | 0.088 |
| Arterial Peripheric disease | 1.04 (0.99-1.08) | 0.107 | 1.04 (0.99-1.09) | 0.161 | 0.99 (0.89-1.11) | 0.882 | 2.06 (0.87-4.85) | 0.098 |
| Peripheral Neuropathy | 1.06 (1.00-1.12) | **0.035** | 1.05 (0.99-1.12) | 0.100 | 0.93 (0.82-1.06) | 0.292 | 2.60 (0.97-7.01) | 0.058 |

Supplementary Table 1: CGM: continuous glucose monitoring. TIR: Time in range, 70-180 mg/dL. TB54: Time below 54 mg/dL. TBR: Time below range, 70 mg/dL. TA250: Time above 250 mg/dL. TAR: Time above range, 180 mg/dL. CV: Glucose variability, defined as the percentage coefficient of variation. GMI: glucose management indicator), is a parameter derived from the measured glucose levels. HbA1c: glycated hemoglobin.

| **Supplementary Table 2. Characteristics of the study sample discriminated by pump or non-pump users** | | | | |
| --- | --- | --- | --- | --- |
|  |  | **Non-Pump Users (n=96)** | | **Pump Users (n=65)** |
| Male sex, % | | | 60 (62.5%) | 37 (56.9%) |
| Age, years | | | 40.3 ± 13.3 | 33.2 ± 12.4 |
| Duration of diabetes, years | | | 17.3 ± 11.6 | 18.3 ± 8.9 |
| Hypertension, % | | | 20 (20.8%) | 10 (15.9%) |
| Dyslipidemia, % | | | 42 (43.8%) | 23 (37.1%) |
| Body mass index, kg/m^2^ | | | 24.9 ± 4.1 | 24.6 ± 4.0 |
| Systolic pressure, mmHg | | | 130.2 ± 16.8 | 121.0 ± 12.1 |
| Diastolic pressure, mmHg | | | 74.3 ± 9.9 | 70.4 ± 9.7 |
| Total cholesterol, mg/dL | | | 165.4 ± 35.1 | 163.3 ± 34.2 |
| LDL cholesterol, mg/dL | | | 96.5 ± 29.5 | 90.1 ± 23.2 |
| HDL cholesterol, mg/dL | | | 55.0 ± 13.2 | 59.7 ± 15.9 |
| Triglycerides, mg/dL | | | 85.7 ± 40.7 | 67.1 ± 31.1 |
| Education | | |  |  |
| Less than hight school graduate, % | | | 1 (3.3 %) | 0 (0.0 %) |
| High school graduate, % | | | 0 (0.0 %) | 1 (2.9 %) |
| Some college education, % | | | 6 (20.0%) | 17 (48.6%) |
| College degree or higher, % | | | 23 (76.7%) | 17 (48.6%) |
| Other antidiabetic drugs | | |  |  |
| SGLT2 inhibitors, % (falta 1 pessoa) | | | 17 (17.7%) | 1 (1.5 %) |
| Metformin, % (falta 1 pessoa) | | | 15 (15.6%) | 2 (3.1 %) |
| GLP-1 analogue, % | | | 4 (4.2 %) | 1 (1.5 %) |
| DPP-4 inhibitors, % (falta1 pessoa) | | | 1 (1.0%) | 0 (0%) |
| Thiazolidinedione, % | | | 0 (0%) | 0 (0%) |
| Sulfonylureas, % | | | 0 (0%) | 0 (0%) |
| Any complication | | | 34 (35.4%) | 18 (27.7%) |
| Microvascular complication | | | 32 (33.3%) | 17 (26.2%) |
| Retinopathy, % | | | 26 (27.1%) | 13 (20.0%) |
| Nephropathy, % | | | 11 (11.5%) | 7 (10.8%) |
| Peripheric neuropathy, % | | | 4 (4.2 %) | 0 (0%) |
| Macrovascular complication | | | 10 (10.4%) | 3 (4.6 %) |
| Ischemic coronary disease, % | | | 3 (3.1 %) | 1 (1.5 %) |
| Cerebrovascular disease, % | | | 4 (4.2 %) | 1 (1.5 %) |
| Peripheral arterial disease, % | | | 5 (5.2 %) | 1 (1.5 %) |
| Heart Failure | | | 0 (0%) | 0 (0%) |

Supplementary Table 2: Categorical variables are presented as counts (percentages). Continuous variables are presented as mean ± standard deviation. LDL: low-density lipoprotein. HDL: High-density lipoprotein. SGLT2: Sodium-glucose Cotransporter-2. GLP-1: Glucagon-like peptide-1. DPP4: Inhibitors of dipeptidyl peptidase 4

| **Supplementary Table 3. HbA1c levels and CGM-metrics discriminated by pump or non-pump users** | | |  |
| --- | --- | --- | --- |
|  | **Non-Pump Users (n=96)** |  | **Pump Users**  **(n=65)** |
| HbA1c, % | 7.5 ± 1.1 | | 7.5 ± 1.2 |
| Amount of time CGM is active, % | 91.4 ± 8.9 | | 89.2 ± 10.0 |
| TIR, % | 57.2 ± 18.3 | | 58.0 ± 15.7 |
| TBR, % | 4.2 ± 4.1 | | 5.9 ± 4.4 |
| TB54, % | 0.9 ± 1.9 | | 1.5 ± 2.2 |
| TAR, % | 38.5 ± 19.1 | | 36.1 ± 16.8 |
| TA250, % | 14.4 ± 13.3 | | 13.1 ± 11.6 |
| CV, % | 37.9 ± 7.6 | | 40.1 ± 7.1 |
| GMI, % | 7.4 ± 0.8 | | 7.3 ± 0.7 |

Supplementary Table 3: Categorical variables are presented as counts (percentages). Continuous variables are presented as mean ± standard deviation. CGM: continuous glucose monitoring. TIR: Time in range, 70-180 mg/dL. TB54: Time below 54 mg/dL. TBR: Time below range, 70 mg/dL. TA250: Time above 250 mg/dL. TAR: Time above range, 180 mg/dL. CV: Glucose variability, defined as the percentage coefficient of variation. GMI: glucose management indicator), is a parameter derived from the measured glucose levels. HbA1c: glycated hemoglobin.

| **Supplementary Table 4. Association of TIR levels (per 1% increase) with diabetes complications** | | | |
| --- | --- | --- | --- |
|  | TIR  (per 1% increase) | |  |
|  | Odds ratio  (95% CI) | P Value |  |
| **Outcomes** |  |  |  |
| Any complication |  |  |  |
| Unadjusted | 0.96 (0.94-0.99) | **0.001** |  |
| Model 1 | 0.96 (0.94-0.99) | **0.012** |  |
| Model 2 | 0.94 (0.90-0.99) | **0.009** |  |
| Microvascular complications |  |  |  |
| Unadjusted | 0.97 (0.95-0.99) | **0.002** |  |
| Model 1 | 0.97 (0.94-1.00) | **0.028** |  |
| Model 2 | 0.95 (0.92-1.00) | **0.029** |  |
| Retinopathy |  |  |  |
| Unadjusted | 0.97 (0.95-0.99) | **0.006** |  |
| Model 1 | 0.97 (0.94-1.00) | **0.047** |  |
| Model 2 | 0.97 (0.94-1.01) | 0.195 |  |
| Nephropathy |  |  |  |
| Unadjusted | 0.98 (0.95-1.01) | 0.234 |  |
| Model 1 | 0.99 (0.96-1.03) | 0.704 |  |
| Model 2 | 0.96 (0.91-1.02) | 0.200 |  |
| Macrovascular complications |  |  |  |
| Unadjusted | 0.96 (0.93-0.99) | **0.019** |  |
| Model 1 | 0.96 (0.91-1.00) | 0.070 |  |
| Model 2 | 0.96 (0.91-1.01) | 0.152 |  |

Supplementary Table 4: Model 1: adjusted to age, sex, and duration of diabetes Model 2: adjusted to age, sex, and duration of diabetes, hypertension and dyslipidemia. CGM: continuous glucose monitoring. TIR: Time in range, 70-180 mg/dL.

**Supplementary Figure 1.**

Number of accounts in LibreView n= 884

N=722

Accounts without data upload

n= 162

N=642

Repeated accounts

n= 80

N=186

No CGM data or active sensor time below 70% at the time of HbA1c analysis

n= 456

Exclusion of other types of diabetes

n= 25

N=161

included

Supplementary Figure 1: Flowchart – Participant recruitment
